# Still mesoendemic onchocerciasis in two Cameroonian community-directed treatment with ivermectin projects despite more than 15 years of mass treatment

**DOI:** 10.1186/s13071-016-1868-8

**Published:** 2016-11-14

**Authors:** Guy-Roger Kamga, Fanny N. Dissak-Delon, Hugues C. Nana-Djeunga, Benjamin D. Biholong, Stephen Mbigha-Ghogomu, Jacob Souopgui, Honorat G. M. Zoure, Michel Boussinesq, Joseph Kamgno, Annie Robert

**Affiliations:** 1 Ministry of Public Health, N°8, Rue 3038 quartier du Lac, Yaoundé, Cameroon; 2 Centre for Research on Filariasis and other Tropical Diseases (CRFilMT), P.O. Box 5797, Yaoundé, Cameroon; 3 Institut de Recherche Expérimentale et Clinique, Faculté de santé publique, Université catholique de Louvain, Clos Chapelle-aux-champs 30 bte B1.30.13 BE-1200, Brussels, Belgium; 4 Institute of Biology of Molecular Medicine, Université Libre de Bruxelles, Rue des professeurs Jeener et Brachet 12 BE-6041 Gosselies, Brussels, Belgium; 5 Faculty of Medicine and Biomedical Sciences, University of Yaoundé 1, P.O. Box 1364, Yaoundé, Cameroon; 6 Molecular and Cell Biology Laboratory, Department of Biochemistry and Molecular Biology, University of Buea, P.O. Box 63, Buea, Cameroon; 7 World Health Organization, African Programme for Onchocerciasis Control (APOC), 01 P.O. Box 549, Ouagadougou 01, Burkina Faso; 8 Institut de Recherche pour le Développement (IRD), IRD UMI 233 TransVIHMI – Université Montpellier – INSERM U1175, 911 avenue Agropolis, P.O. Box 64501, 34394 Montpellier Cedex 5, France

**Keywords:** Onchocerciasis, Ivermectin, Persistence, Elimination, Bafia, Yabassi, Cameroon

## Abstract

**Background:**

After more than a decade of community-directed treatment with ivermectin (CDTI) in Centre and Littoral Regions of Cameroon, onchocerciasis endemicity was still high in some communities according to the 2011 epidemiological evaluations. Some corrective measures were undertaken to improve the CDTI process and therefore reduce the burden of the disease. The objective of the present study was to assess the progress made towards the elimination of onchocerciasis in the Centre 1 and Littoral 2 CDTI projects where the worst performances were found in 2011. To this end, a cross-sectional survey was conducted in April 2015 in eight communities in two health districts (HD), Bafia in Centre 1 and Yabassi in Littoral 2, chosen because assessed at baseline and in 2011. All volunteers living for at least five years in the community, aged five years or more, underwent clinical and parasitological examinations. Individual compliance to ivermectin treatment was also assessed. Analyses of data were weighted proportionally to age and gender distribution in the population.

**Results:**

In the Bafia and Yabassi HD, 514 and 242 individuals were examined with a mean age of 35.1 (standard deviation, SD: 20.7) and 44.6 (SD: 16.3) years, respectively. In the Bafia HD, the weighted prevalences varied from 24.4 to 57.0 % for microfilaridermia and from 3.6 to 37.4 % for nodule presence across the surveyed communities. The community microfilarial load (CMFL), expressed in microfilariae/skin snip (mf/ss), significantly dropped from 20.84–114.50 mf/ss in 1991 to 0.31–1.62 mf/ss in 2015 in all the surveyed communities. In the Yabassi HD, the weighted prevalences varied from 12.3 to 59.3 % for microfilaridermia and from 1.5 to 3.7 % for nodule presence across the surveyed communities, while a significant drop was observed in CMFL, from 20.40–28.50 mf/ss in 1999 to 0.48–1.74 mf/ss in 2015. The 2014 weighted therapeutic coverage of participants varied from 65.8 % (95 % CI: 58.4–73.2) in Yabassi HD, to 68.0 % (95 % CI: 63.3–72.7) in Bafia HD, with important variations among communities.

**Conclusions:**

After more than 15 years of CDTI, onchocerciasis is still mesoendemic in the surveyed communities. Further studies targeting therapeutic coverage, socio-anthropological considerations of CDTI implementation and entomological studies would bring more insights to the persistence of the disease as observed in the present study.

**Electronic supplementary material:**

The online version of this article (doi:10.1186/s13071-016-1868-8) contains supplementary material, which is available to authorized users.

## Background

Onchocerciasis, better known as river blindness, is a debilitating insect-borne parasitic disease caused by *Onchocerca volvulus* and transmitted via the bites of blackflies of the genus *Simulium*. The larvae and pupae of the latter develop in fast-flowing and well-oxygenated streams and rivers. The prevalence of infection and disease in a community is therefore related to riverine breeding sites of the vector. The disease is endemic in 30 African countries, in Yemen and in localized foci in four Latin American countries. About 120 million people are at risk worldwide, with 99 % of them living in Africa, and 37 million were infected in 1995, when the African Programme for Onchocerciasis Control (APOC) was launched [1, 2].

Adult *O. volvulus* average lifespan is estimated to 10–15 years [3, 4]. Individual female worms produce daily thousands of microfilariae whose lifespan varies from 12 to 18 months. By invading the host dermis and eyes, live microfilariae interact with the host immune system, while dead microfilariae induce inflammatory responses, causing a variety of skin and ocular symptoms [5–7]. Infection leads to severe skin damage with unrelenting itching, visual impairment and blindness. Irreversible onchocercal blindness is ranked as the world’s second leading infectious cause of preventable blindness after trachoma [8]. Besides its clinical impact, river blindness also has an important socio-economic impact on affected populations. It creates stigma [9] and generates and perpetuates poverty. Fear of the disease often prompted people to abandon fertile lands which in turn led to an increase in poverty and famine, making the disease a major obstacle to socioeconomic development. The agricultural productivity is therefore hindered, generating massive economic losses and imposing a disproportionate disease burden in poor rural communities [10].

Ivermectin is currently the only known effective and safe drug used for mass treatments against onchocerciasis. However, since this drug has a limited macrofilaricidal activity, treatments must be repeated for more than 15 years. The control of onchocerciasis has been quite successful with the implementation of Community Directed Treatment with Ivermectin (CDTI). This strategy, proposed by the World Health Organization (WHO) through APOC, has significantly improved Ivermectin treatment coverage [11–13]. While this drug has been administrated twice or four times a year in the small and well-delineated endemic communities of the Americas, single doses have been given yearly in the much larger foci in African endemic countries [14, 15]. As a consequence, the programmes in the Americas are highly successful and are attaining the onchocerciasis elimination point [16–20], while this disease remains a public health problem in Africa. Nevertheless, new evidence points exist towards the possibility of successful elimination of river blindness in Africa using ivermectin solely [21, 22]. Indeed, a spectacular decrease in microfilaridermia prevalence below 1 % was reported in Mali and Senegal [22], as well as in some CDTI-project in the APOC countries [23, 24]. However, despite more than 20 years of disease control, onchocerciasis remains a major concern for several endemic countries, including Cameroon [25]. Indeed, recent WHO/APOC surveys conducted in 2011 revealed onchocerciasis prevalences above 60 % in the Centre 1, Littoral 2 and West CDTI-projects in Cameroon [24]. The reasons for the persistence of the infection are yet to be elucidated, and it is not clear whether this is due to low treatment coverage, systematic non-compliance of a proportion of the population, or suboptimal response of the parasite to ivermectin [26–28]. These poor results led to the implementation of some corrective measures (frequent and regular supportive supervision, data quality audit, community self-monitoring and a better drug management) by the National Onchocerciasis Control Programme to improve the CDTI performances.

The objective of the present survey was, therefore, to assess the progress made towards the elimination of onchocerciasis in the Centre 1 and Littoral 2 CDTI projects where the worst performances were found in 2011.

## Methods

### Study area and selection of communities

The present study was conducted in the Bafia (4°45′00″N, 11°14′00″E) and Yabassi (4°27′16″N, 9°57′56″E) health districts (HD), belonging to the Centre 1 and Littoral 2 CDTI projects, respectively.

The Bafia HD is located in the Mbam and Inoubou Division (Centre Region), at 120 km north from Yaoundé, the political capital of Cameroon. In 2014, its population was 226,073 inhabitants, based on a census conducted by community-directed distributors (CDD). The altitude of this region varies from 1,100 to 1,300 m. It is a forest-savanna transition zone, irrigated by many fast-flowing rivers including Sanaga and its tributaries, as well as the Mbam and Noun rivers. The main activities of inhabitants are agriculture (mainly cocoa), fishing and sand mining.

The Yabassi HD is located in the Nkam Division (Littoral Region), at 100 km north-east from Douala, the economic capital of the country. According to the 2014 CDD census, its population was 21,459 inhabitants. The relief is undulating, showing an alternation of valleys and plains. Altitude of the region varies from 10 to 800 m. This district is irrigated by many fast-flowing rivers comprising Nkam, Dibamba, Mabombé, Njanga and Mahé which are favorable to blackfly breeding. The vegetation is mainly dense humid forest. Agriculture is the main activity, interesting at least 60 % of inhabitants.

### Baseline and follow-up surveys

**Fig. 1:**
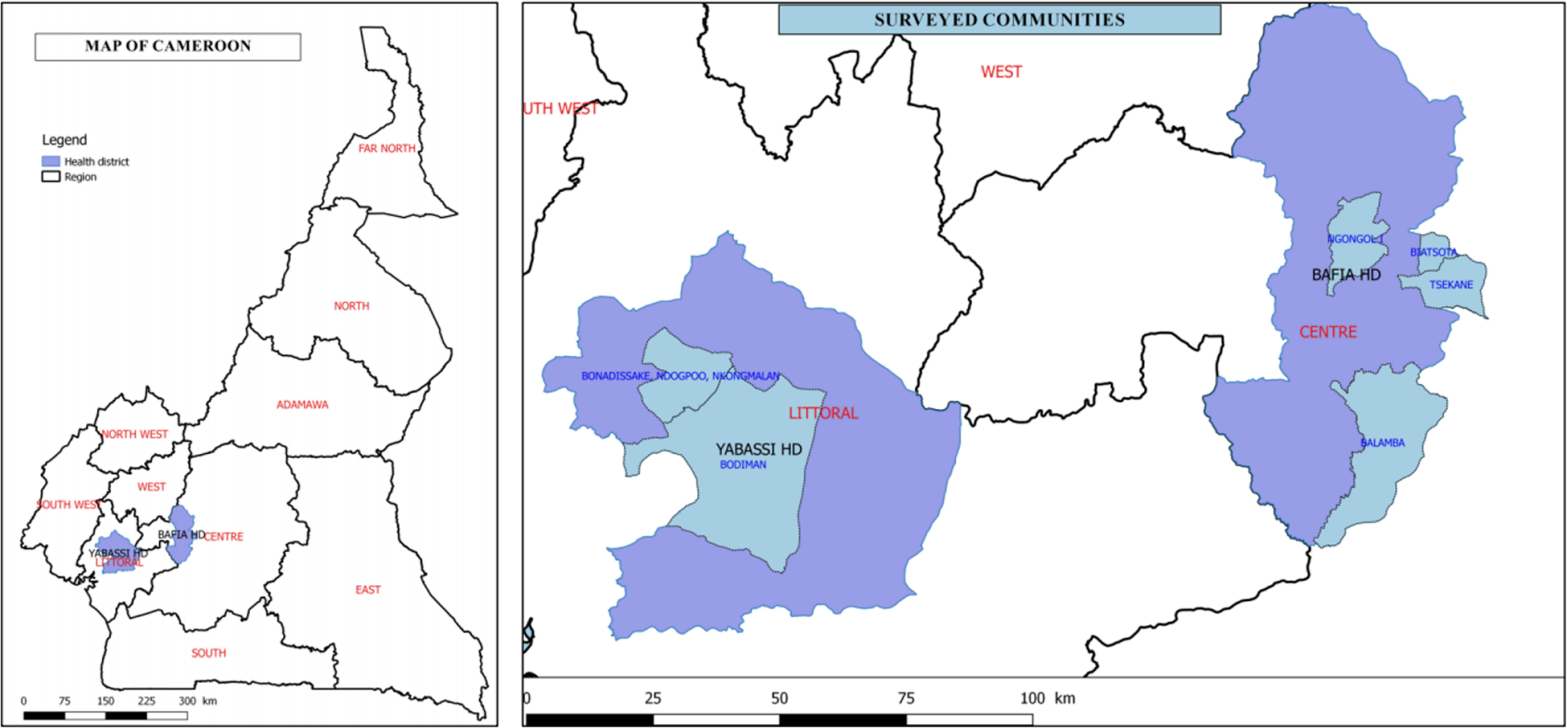
Map of Cameroon showing the study areas and communities surveyed

Surveyed communities were selected according to the availability of baseline data and the results of the 2011 follow-up survey carried out by the National Onchocerciasis Control Programme (NOCP) with the support of APOC (NOCP, unpublished report). The prevalence and intensity of infection (nodules and microfilaridermia) had also been assessed in 1991–1993 by an IRD-Centre Pasteur du Cameroun team in seven communities of the Bafia HD [29], and in 1999 in seven communities of the Yabassi HD (Kamgno, unpublished report). From these 14 communities, four (Balamba II, Biatsota, Ngongol and Tsékané) in the Bafia HD and four (Bodiman, Bonadissake, Ndogpoo and Nkongmalan) in the Yabassi HD, with the highest microfilaridermia prevalence and/or community microfilarial load (CMFL), were selected for the present survey (Tables 1 and 2; Fig. 1).

**Table 1:** Onchocerciasis burden in four communities of Bafia health district at baseline and during the 2011 survey, after 11 years of CDTI

Village	Baseline data (1991–1993)			Follow-up survey (2011)		
	*N*	Weighted mf prevalence (%)	CMFL (mf/ss)	*N*	Weighted mf prevalence (%)	CMFL (mf/ss)
Tsékané	150	90.8	20.84	204	47.1	1.44
Balamba II	167	83.7	26.40	212	61.0	4.72
Ngongol I	129	92.8	90.31	237	64.0	2.54
Biatsota	150	94.0	114.50	227	67.6	3.26

*Abbreviations*: *N* number examined, *mf* microfilaria, *CMFL* community microfilarial load (microfilariae/skin snip)

**Table 2:** Onchocerciasis burden in four communities of Yabassi health district at baseline and during the 2011 survey

Village	Baseline data (1999)			Follow-up survey (2011)		
	*N*	Weighted mf prevalence (%)	CMFL (mf/ss)	*N*	Weighted mf prevalence (%)	CMFL (mf/ss)
Bonadissake	58	79.3	20.4			
Ndogpoo	39	87.2	24.2			
Nkongmalan	84	92.2	28.5			
Bodiman	81	90.1	26.5	112	65.2	2.96

*Abbreviations*: *N* number examined, *mf* microfilaria, *CMFL* community microfilarial load (microfilariae/skin snip)

### History of mass treatment with ivermectin

**Fig. 2:**
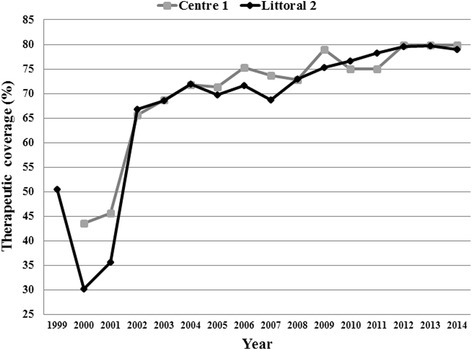
Therapeutic coverage in Centre 1 and Littoral 2 CDTI projects

In the Bafia HD, a clinical trial was conducted between 1994 and 1998, with mass treatment carried out in some communities [30, 31]. However, according to the NOCP, CDTI started in 1999 and in 2000 in Littoral 2 and Centre 1 projects, respectively [32]. So these communities received 16 rounds and 15 rounds of mass drug administration, respectively. The therapeutic coverage achieved in each project is illustrated in Fig. 2. Even though the two projects achieved the treatment coverage threshold for control (65 %) during the last 13 years, none of them reached 80 % treatment coverage, the threshold for elimination, although a significant improvement has been achieved since 2012.

### Study design and patients

A cross-sectional survey was conducted in April 2015 in eight communities of the Bafia and Yabassi health districts, nine months after the previous annual ivermectin distribution. All individuals, permanent residents or who had already lived for at least five years in the community and aged five years or more were eligible for this survey. All volunteers underwent clinical and parasitological examinations. In addition, individual compliance to ivermectin treatment was also assessed.

### Clinical examination

Each participant was examined for skin disease and nodules. The searched cutaneous signs were depigmentation, onchodermatitis (acute, chronic and lichenified), hanging groin and skin atrophy. Participants were scored as ‘positive’ or ‘negative’ to each cutaneous sign. Nodule palpation was performed in a closed but well-illuminated room. Qualified and certified staff performed the palpation on partially disrobed participants while paying particular attention to bony prominences of the torso, iliac crests and upper trochanter of the femurs. Onchocercal nodules were identified clinically as mobile masses beneath the skin, firm and painless [33–35]. Results were ranked as ‘positive’ or ‘negative’, and if positive, the number and location of all palpable onchocercal nodules were recorded.

### Parasitological examination

Immediately following the nodule palpation, two skin snips were taken from each posterior iliac crest using a 2 mm corneoscleral punch (Holth-type). The skin samples were immediately placed, separately, into wells of microtitration plates containing a sterile normal saline solution. After 24 h incubation at room temperature, the skin snips were removed and the fluid from each well was examined under low magnification (40×) by trained laboratory technicians [36]. For positive results, the microfilariae were counted and the individual microfilarial densities were expressed as the arithmetic mean number of microfilariae in the two skin snips (mf/ss).

### Assessment of the individual compliance to ivermectin mass treatment

Individual compliance to ivermectin mass treatment was assessed by asking each participant if he/she swallowed ivermectin tablets during each of the previous five years (including the last CDTI-campaign). Ivermectin tablets were presented to the participants to make sure that the interview was targeting the right treatment. The participant answers were recorded on an individual form as “yes” or “no” for each of the last five years.

### Data analysis

All relevant data (clinical signs, nodule, mf count, and compliance to ivermectin mass treatment) were recorded into a purpose-built Microsoft Access database and subsequently exported into STATA 13 for statistical analysis. All analyses were weighted proportionally to age- and gender distribution in the population, according to 2015 national demographic data projections [37]. Cutaneous signs, nodules and microfilaridermia prevalences were expressed as the proportion of infected or affected individuals with the 95 % exact confidence interval (CI). Each community was classified according to the prevalence of microfilaridermia as hypoendemic (prevalence ˂ 35 %), mesoendemic (35 % ≤ prevalence ˂ 60 %) or hyperendemic (prevalence ≥ 60 %) [38]. When the microfilarial count was positive, the intensity of infection was computed as the arithmetic mean and its 95 % CI. The community microfilarial load (CMFL), defined as the geometric mean number of microfilariae per skin snip among adults aged 20 years or more, was calculated using a log (x + 1)-transformation:

$$ \mathrm{CMFL}={\mathrm{e}}^{\frac{1}{\mathrm{N}}{\displaystyle \sum } \ln \left(\mathrm{x}+1\right)}-1 $$

where x is the individual microfilarial density, and N the total number of individuals aged 20 years and above. Proportions were compared using Chi-square tests. Mean intensities of infection were compared between age- and gender subgroups using ANOVA with *F*-tests or *t*-tests. A *P*-value < 0.05 was considered as statistically significant.

## Results

In the two HD, 514 and 242 individuals were examined. Mean ages were 35.1 (SD: 20.7) and 44.6 (SD: 16.3) years, respectively. The sex ratio was slightly female-biased in the Bafia HD (female proportion of 51.8 %), but was male-biased in the Yabassi HD (female proportion equal to 45.9 %).

### Prevalence and intensity of infection

In Bafia HD, weighted prevalences varied from 24.4 to 57.0 % for microfilaridermia and 3.6 to 37.4 % for nodule prevalence across the surveyed communities. Men were more affected than women, both for microfilaridermia (47.5 *vs* 36.5 %; *χ* ^2^ = 6.4, *df* = 1, *P* = 0.03) and nodule presence (27.1 *vs* 10.5 %; *χ* ^2^ =23.8, *df* = 1, *P* < 0.001). The communities Biatsota and Ngongol I had the highest burden, both for microfilaridermia and nodule prevalence, and were ranked as mesoendemic according to the microfilaridermia prevalence. Balamba II and Tsékané were ranked as hypoendemic although still presenting with high prevalences after 15 years of treatment (Table 3). Across age groups, no significant differences in microfilaridermia prevalence were observed, while nodule prevalence increased with age. Arithmetic mean of microfilaridermia among positive cases was higher in younger participants (9–19 years) and decreased with age, thus displaying an opposite trend to the one observed for nodule prevalence (Table 4).

**Table 3:** Parasitological load and clinical signs of onchocerciasis within each community in the present survey of 2015

District and community		Microfilaridermia examination			Clinical examination weighted prevalence (%)	
	*N*	Weighted prevalence (%)	CMFL (mf/ss)	Status	Nodule	Cutaneous sign
Bafia						
Balamba II	138	24.4	0.51	Hypoendemic	3.6	1.1
Biatsota	94	45.8	1.04	Mesoendemic	37.4	7.0
Ngongol I	186	57.0	1.62	Mesoendemic	23.9	1.5
Tsékané	96	26.4	0.31	Hypoendemic	7.2	3.0
Total	514	41.6		Mesoendemic	18.1	2.3
Yabassi						
Bodiman	64	59.3	1.39	Mesoendemic	3.7	2.8
Bonadissake	41	12.3	0.48	Hypoendemic	1.6	22.4
Ndogpoo	80	46.7	1.10	Mesoendemic	1.5	10.9
Nkongmalan	57	47.7	1.74	Mesoendemic	3.5	4.6
Total	242	43.8		Mesoendemic	2.7	9.3

*Abbreviations*: *N* number examined, *mf* microfilaria, *CMFL* community microfilarial load (microfilariae/skin snip)

**Table 4:** Microfilaria and nodule status across age groups in the present survey of 2015

District	Age categories (years)		Microfilaria status			Nodule status		
		*N*	Mf+ (*n*)	Weighted prevalence (%)^a^	Weighted mean ± SD of mf in carriers	Nod+ (*n*)	Weighted prevalence (%)^a^	Weighted mean ± SD of nodules in carriers
Bafia	5–9	56	19	34.0	20.5 ± 27.7	9	16.1	1.7 ± 0.7
	10–19	94	47	50.0	24.9 ± 47.6	9	9.6	2.2 ± 1.4
	20–29	76	32	41.5	10.9 ± 22.4	12	14.9	2.1 ± 1.1
	30–39	70	28	40.0	13.8 ± 24.0	23	32.5	2.6 ± 2.1
	40–49	69	24	34.7	13.5 ± 24.6	21	30.4	2.6 ± 2.3
	50–59	70	26	37.2	6.6 ± 9.3	17	24.5	2.8 ± 2.0
	60+	79	20	25.4	7.6 ± 12.5	24	30.0	2.5 ± 2.6
	Total	514	196	41.6	17.9 ± 35.5	115	18.1	2.4 ± 1.8
Yabassi	5–9	0	–	–	–	–	–	–
	10–19	14	7	50.0	21.4 ± 21.3	0	0	0
	20–29	35	13	36.6	16.5 ± 29.3	0	0	0
	30–39	47	23	48.9	16.1 ± 26.5	1	2.1	1
	40–49	48	17	35.4	5.1 ± 6.9	3	6.3	1.7 ± 0.6
	50–59	51	28	54.9	11.2 ± 19.4	4	8.0	2 ± 0.8
	60+	47	21	44.7	7.3 ± 12.5	2	4.1	2.5 ± 0.7
	Total	242	109	43.8	14.2 ± 22.6	10	2.7	1.8 ± 0.7

*Abbreviations*: *N* number examined, *mf* microfilaria, *Mf+ (n)* number with microfilaria positive skin snip, *Nod+ (n)* number of nodule carriers, *SD* standard deviation

^a^In each age category, the indices were adjusted on sex; in the total populations, indices were adjusted on sex and age

**Fig. 3:**
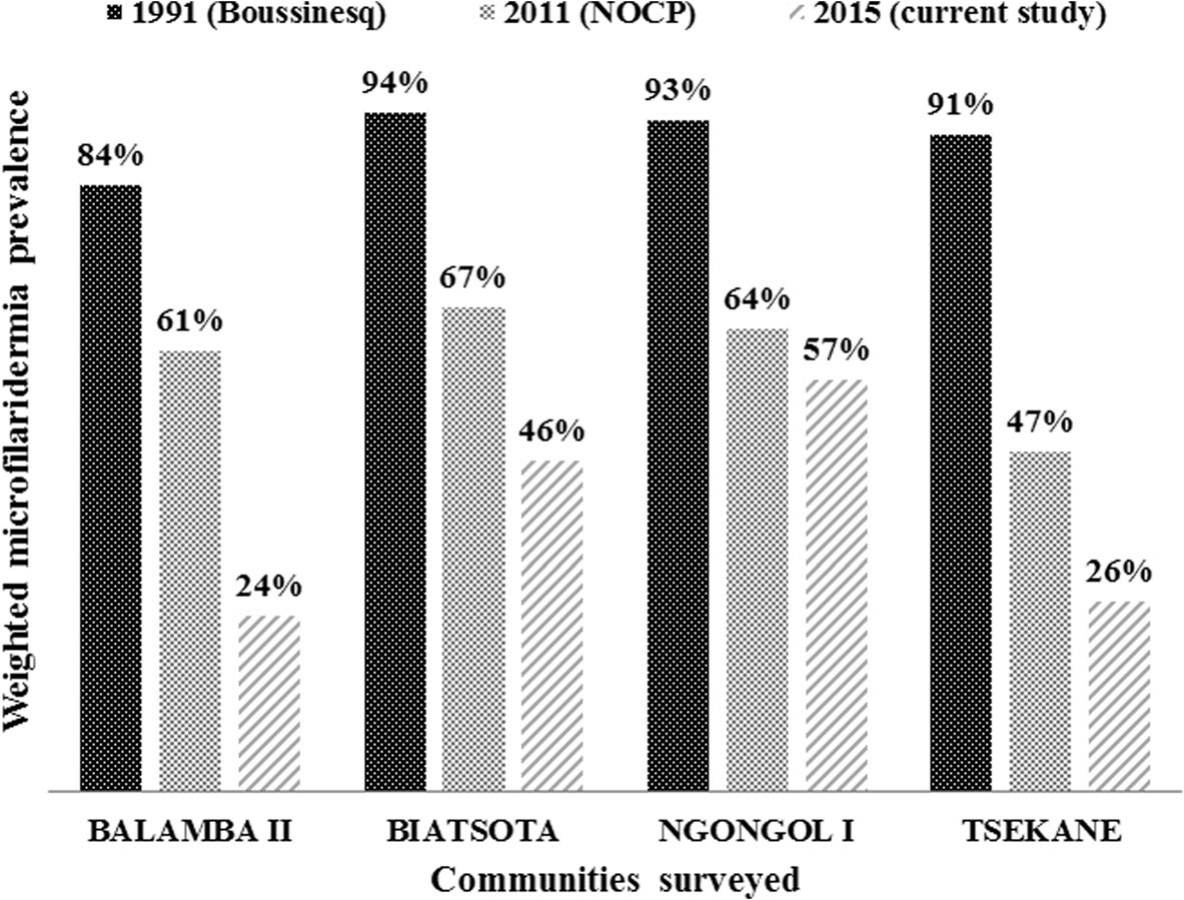
Comparison of weighted microfilaridermia prevalences at baseline, 1991, with follow-up surveys in 2011 and 2015, in the communities surveyed in the Bafia health district

**Fig. 4:**
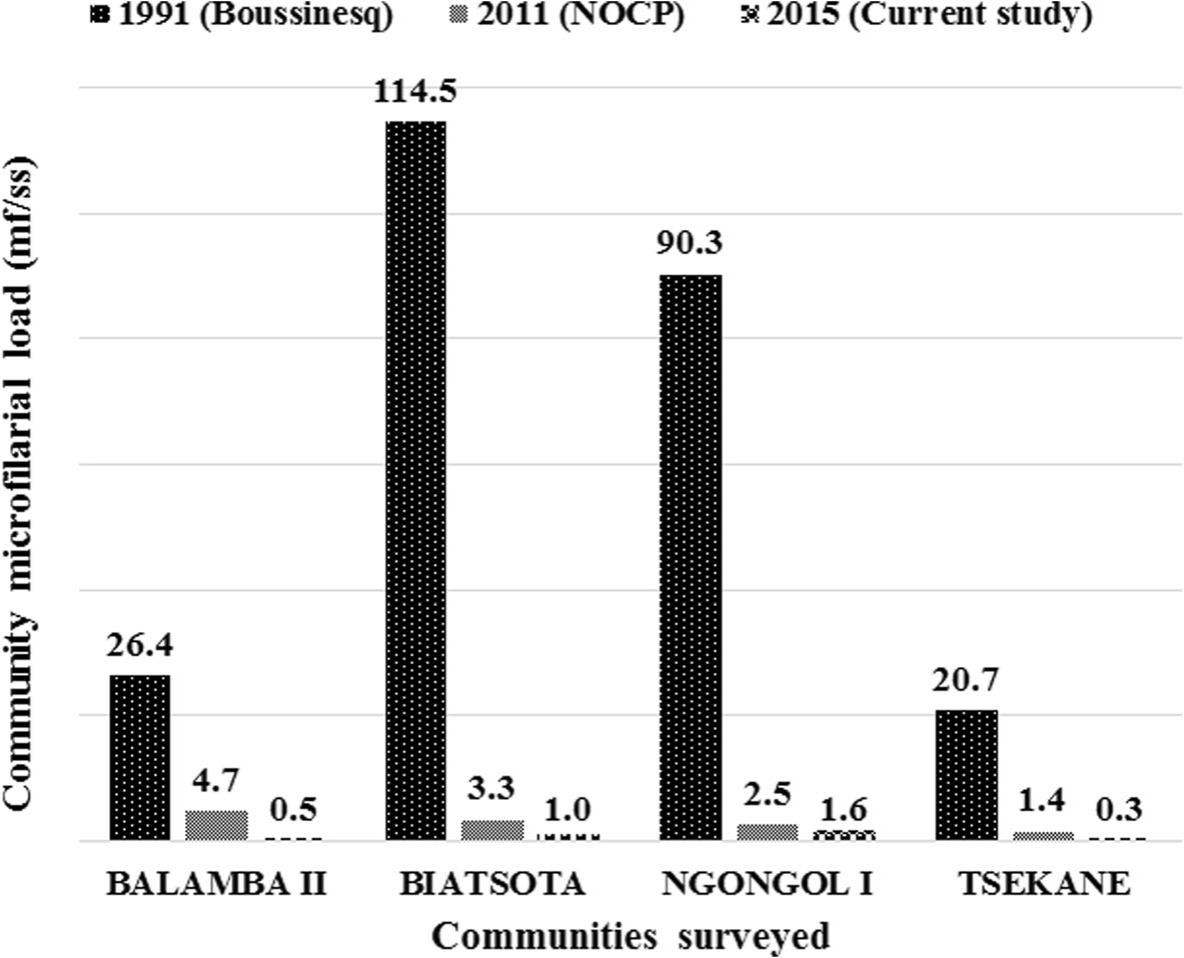
Comparison of community microfilarial load at baseline, 1991, with follow-up surveys in 2011 and 2015, in the communities surveyed in the Bafia health district. *Abbreviations*: mf, microfilaria; ss, skin snip

A significant reduction in prevalence was observed in all communities when comparing the present data to baseline data (*χ* ^2^ = 222.6, *df* = 9, *P* < 0.001) (Fig. 3). The CMFL in all the surveyed communities also significantly dropped by 98-99 % from 20.84–114.50 mf/ss in 1991 to 0.31–1.62 mf/ss in 2015 (ANOVA: *F*_(1,3)_ = 131.6, *P < 0.001*) (Fig. 4).

In Yabassi HD, weighted prevalences varied from 12.3 to 59.3 % for microfilaridermia and from 1.5 to 3.7 % for nodules. Men were more affected than women, both for microfilaridermia (48.7 *vs* 38.4 %; *χ* ^2^ =2.6, *df* = 1, *P* = 0.17) and nodule presence (4.5 *vs* 0.7 %; *χ* ^2^ =3.3, *df* = 1, *P* = 0.07); however, the differences were not statistically significant. Onchocercal skin lesions were more frequent in two communities (Bonadissake and Ndogpoo) where lower limb depigmentation, acute and chronic onchodermatitis were found (Table 3). The arithmetic mean of microfilarial load decreased with age while the mean number of nodules increased (Table 4).

**Fig. 5:**
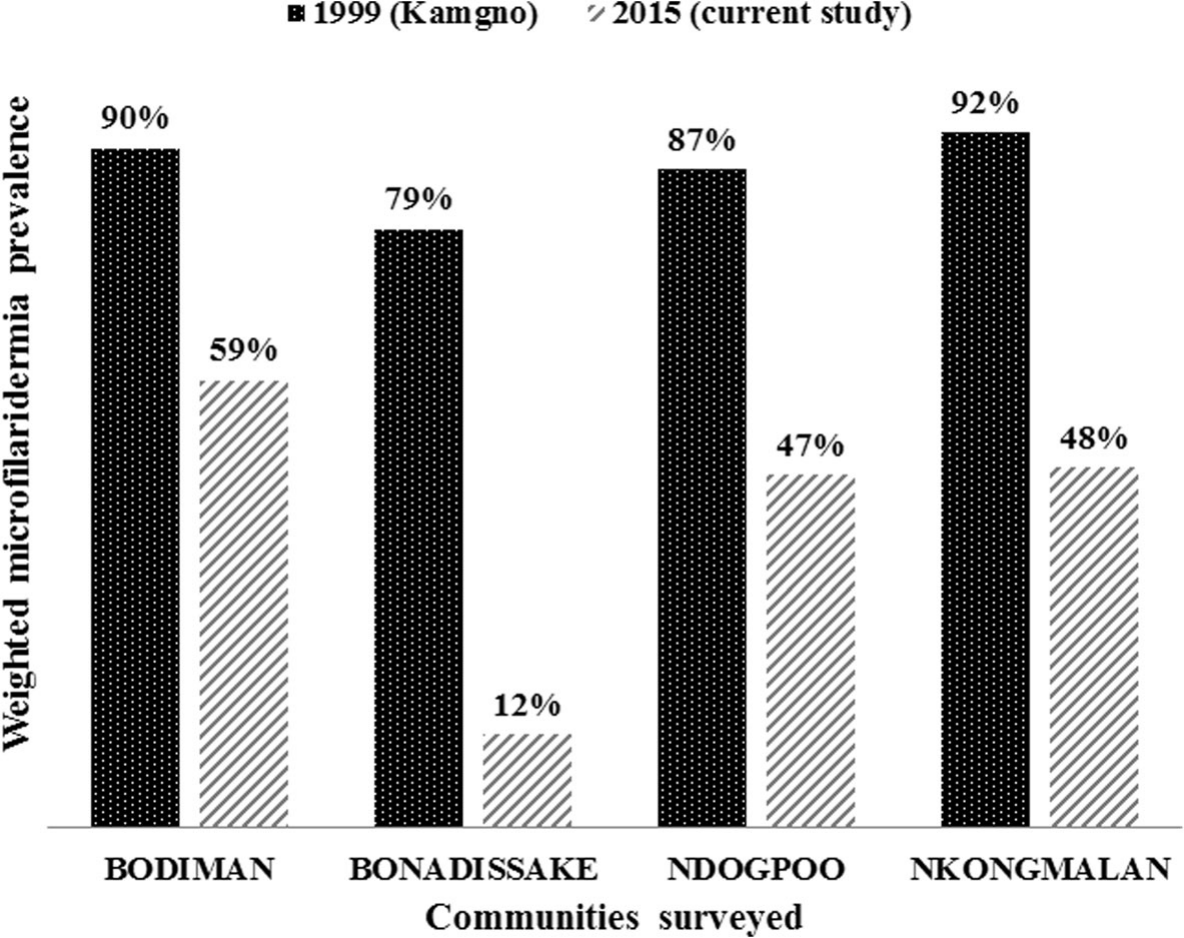
Comparison of weighted microfilaridermia prevalences at baseline, 1999, with follow-up surveys in 2015, in the communities surveyed in the Yabassi health district

**Fig. 6:**
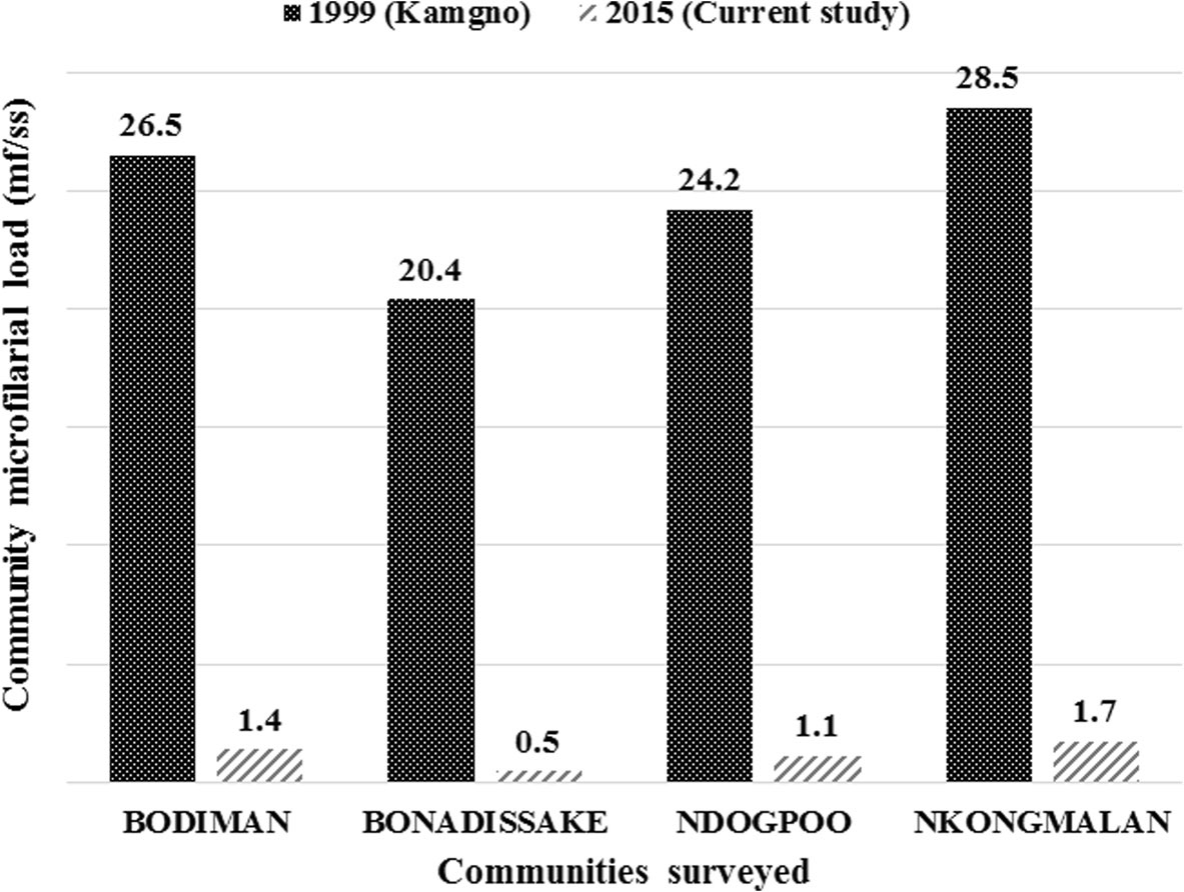
Comparison of community microfilarial load at baseline, 1999, with follow-up surveys in 2015, in the communities surveyed in the Yabassi health district. *Abbreviations*: mf, microfilaria; ss, skin snip

**Fig. 7:**
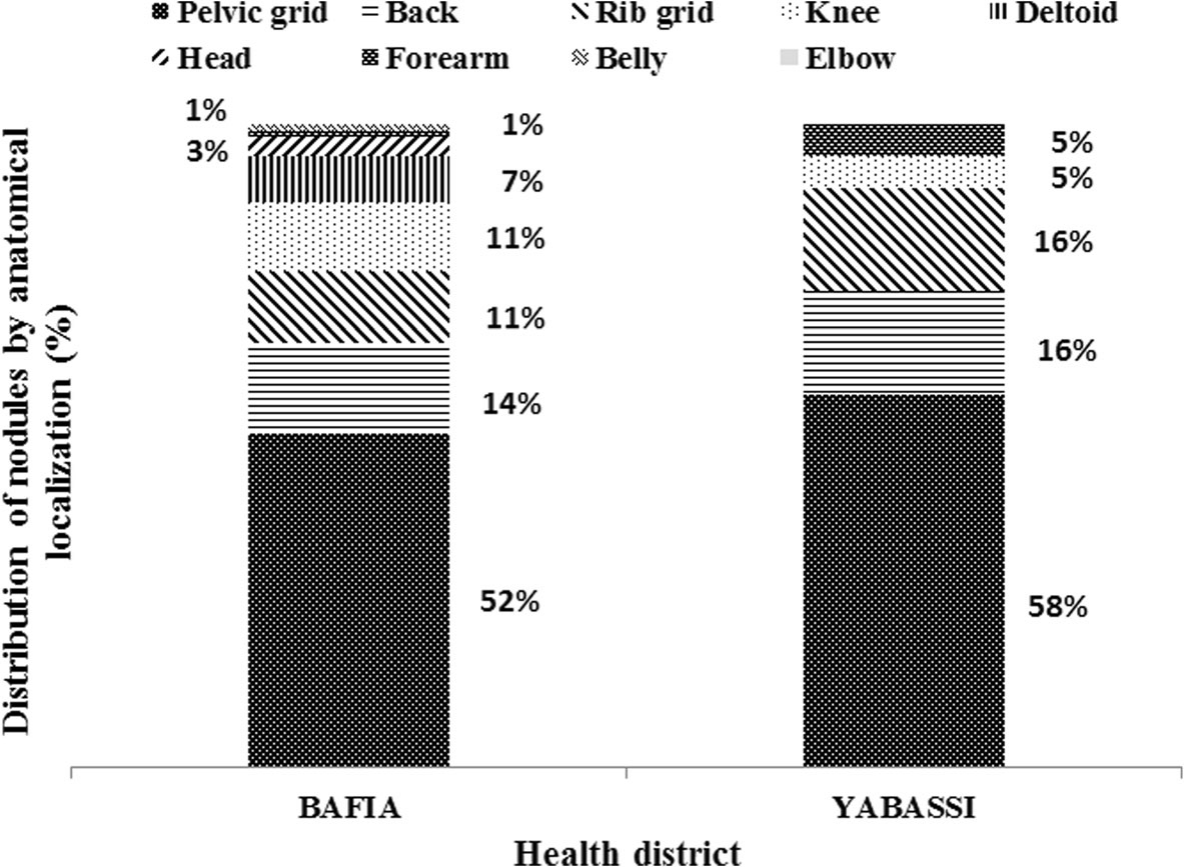
Distribution of nodules according to the anatomical localization in carriers

A significant reduction in prevalence was found in all communities when comparing baseline to the follow-up data in 2015 (*χ* ^2^ = 107.6, *df* = 9, *P* < 0.001) (Fig. 5). A significant drop in CMFL by 94–98 %, was also observed, from 20.40–28.50 in 1999 to 0.48–1.74 mf/ss in 2015 (ANOVA: *F*_(1,3)_ = 1554.7, *P < 0.001*) (Fig. 6). Most palpable nodules were localized in the pelvic grid, the back, the chest and the knee with half of them found in the pelvic grid (Fig. 7).

### Compliance to ivermectin mass treatments in surveyed communities

**Fig. 8:**
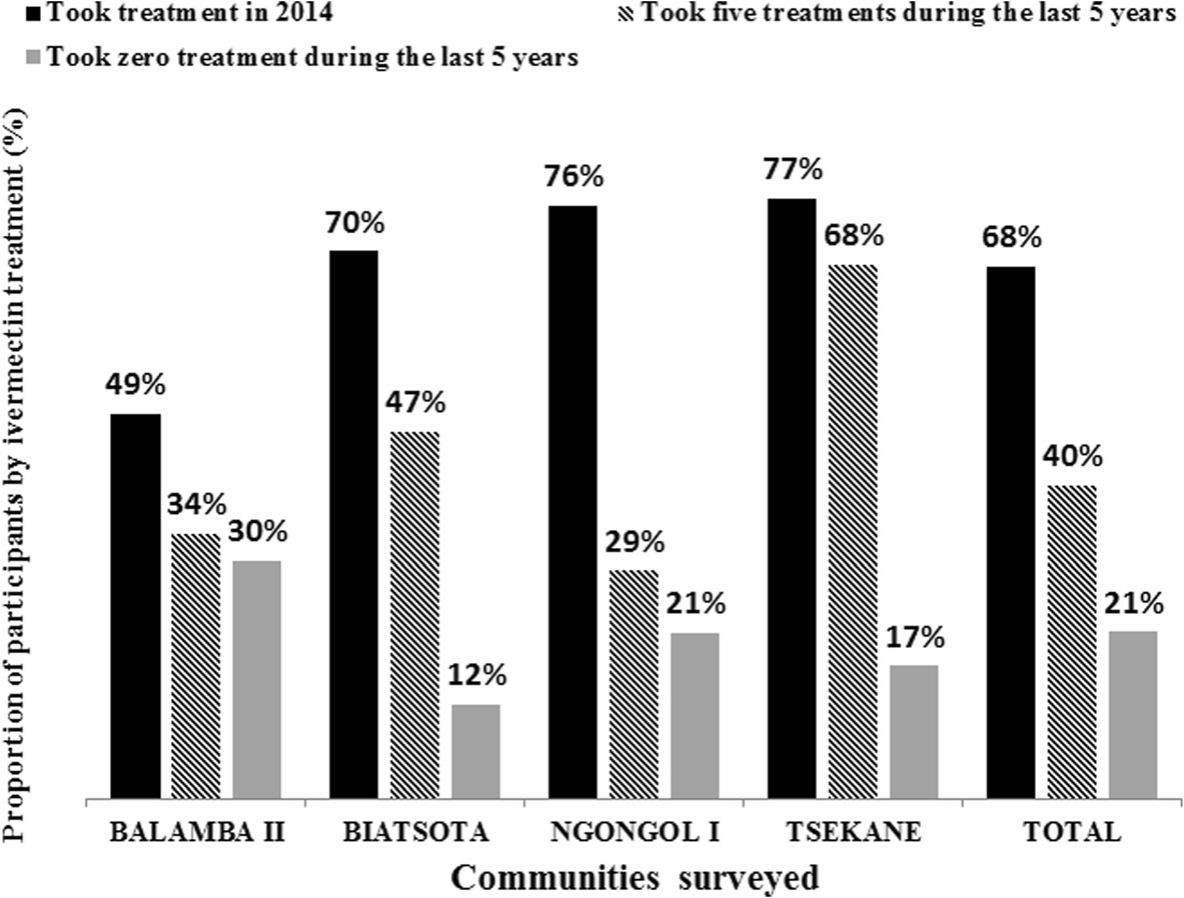
Proportion of participants according to ivermectin treatment in the Bafia health district

**Fig. 9:**
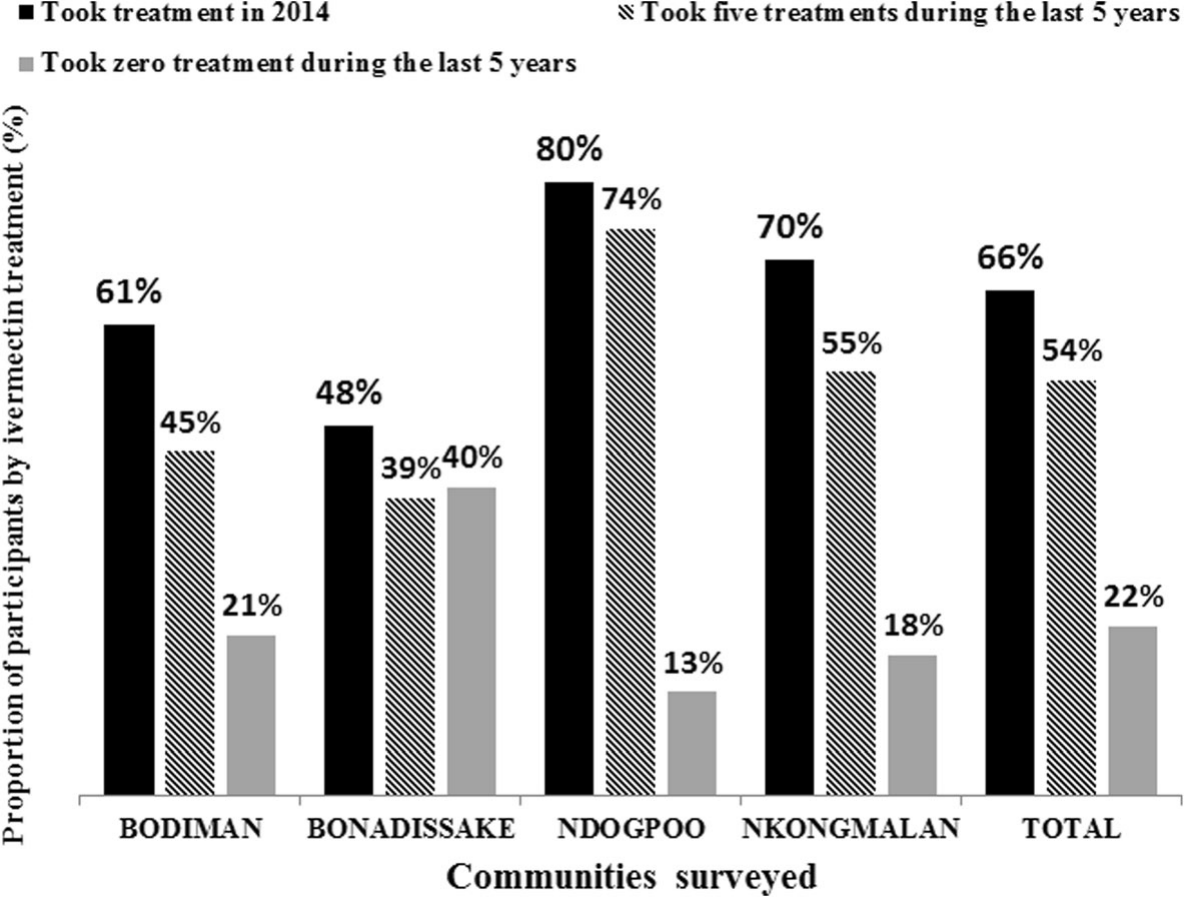
Proportion of participants according to ivermectin treatment in the Yabassi health district

**Fig. 10:**
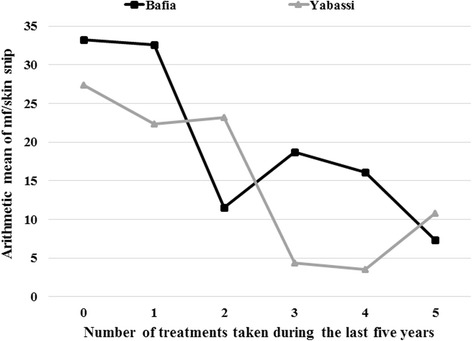
Arithmetic mean of microfilaria per skin snip according to the number of ivermectin treatment taken during the last five years among carriers in Bafia and Yabassi health districts

The weighted therapeutic coverage in 2014 varied from 65.8 % (95 % CI: 58.4–73.2) to 68.0 % (95 % CI: 63.3–72.7) in the surveyed communities from Yabassi HD and Bafia HD, respectively, with important variations across communities. A variation was also observed in the compliance to ivermectin over five years, from 39.9 % (95 % CI: 35.2–44.6) in Bafia HD to 54.0 % (95 % CI: 46.6–61.4) in Yabassi HD. The adherence to treatment increased significantly with age since more than 75 % of participants aged 40 years and above declared having taken the treatment each year during the last five years, whereas only 31 % of individuals aged 10–29 years did so (*χ* ^2^ = 234.6, *df* = 30, *P* < 0.001). More than one out of five participants in Bafia HD (21.3 %; 95 % CI: 17.1–25.5) and Yabassi HD (22.0 %; 95 % CI: 15.3–28.6) declared that they had not taken the treatment during the last five years (Figs. 8 and 9). The microfilaridermia prevalence was associated with treatment compliance in Bafia HD (*χ* ^2^ = 14.0, *df* = 5, *P*= 0.016), but not in Yabassi HD (*χ* ^2^ = 10.5, *df* = 5, *P* = 0.062), whereas its intensity was significantly negatively associated with compliance to treatment in both districts (ANOVA: *F*_(5, 299)_ = 5.95, *P* < 0.001) as shown in Fig. 10.

## Discussion

The objective of this study was to assess the progress made towards the elimination of onchocerciasis in the Centre 1 and Littoral 2 CDTI projects where high prevalence and intensity of infection were found in 2011. In 2015, after more than 15 years of mass treatment, we observed a reduction in microfilaridermia prevalence, but values were still above the expected level (≤ 20 % for a pretreatment endemicity ≥ 80 % assuming a therapeutic coverage of 65 %) [39, 40]. Indeed, onchocerciasis is still mesoendemic in many surveyed communities especially in those closest to fast-flowing rivers. Blackfly breeding sites located a few kilometres from Biatsota, Ngongol I and Bodiman contribute to maintain high vector densities and continuing transmission as was previously demonstrated for first-line communities [29]. The same communities had the highest prevalences of nodules in the two health districts. Furthermore, the high microfilaridermia and nodule prevalences observed in less than 10-year-old children, born after the launch of the programme, are a strong evidence of ongoing active transmission which could later be confirmed by entomological studies. In addition to the parasite burden, the persistence of the disease can also be explained by the low adherence to treatment as only 40 and 54 % of participants in Bafia HD and Yabassi HD, respectively, declared having taken five treatments during the last five years. At the same time, about 20 % of the participants had taken no treatment during that period. With the observed treatment coverage, the likelihood for the programme to achieve elimination of the disease by 2025 is quite low. Even though this survey was conducted nine months after the previous treatment, we believe that our results regarding the therapeutic coverage and the compliance are reliable because ivermectin tablets are special, by both their physical presentation (small and white) and their delivery strategy. So, the likelihood to confuse them with other drugs or interventions is quite low. Previous studies have reported accurate recall of populations when compared to the data from CDTI collected in treatment registry [41–43].

Despite the high mf prevalences observed in both districts, the intensity of infection had dramatically decreased in 2015, with CMFL below 2 mf/ss in all communities, as compared to baseline data. Even when compared to 2011, the CMFL dropped by half in almost all surveyed communities. Furthermore, the arithmetic mean number of microfilariae per skin snip among positive cases was found to be related to compliance to treatment. Thus it could be hypothesized that ivermectin would enable to eliminate onchocerciasis in the study areas if CDTI were properly implemented as demonstrated in Mali and Nigeria [44, 45]. An improvement in therapeutic coverage is a major requirement in these areas with high baseline intensities of infection.

As observed in previous studies, males were more affected than females probably due to their socioeconomic activities [46–48]. In addition, due to their prolonged exposition to the bites of blackflies, the oldest participants have more nodules than their youngest counterparts; meanwhile their adherence to treatment could explain their lowest intensity of infection. This adherence is probably motivated by their awareness about the consequences of the disease.

The localization of nodules was comparable to previous studies [33–35, 49], especially in Biatsota and Ngongol I. Onchocercal skin disease was more frequent in two communities (Bonadissake and Ndogpoo) where lower limb depigmentation, acute and chronic onchodermatitis were found.

## Conclusions

After more than 15 years of CDTI, onchocerciasis is still mesoendemic in the communities surveyed as part of this study. Additional efforts should be made to improve the CDTI therapeutic coverage in all communities so as to greatly reduce the prevalence and the microfilarial densities in order to eliminate this debilitating disease in these two CDTI-projects. Further studies targeting community therapeutic coverage, CDTI process and entomological studies would provide better insights into our understanding of the persistence of the disease as described in this study.

## Additional file

Additional file 1: Datasets of Bafia and Yabassi health districts supporting this study. (XLSX 114 kb)

## Abbreviations

APOC: African Programme for Onchocerciasis Control
ARES-CCD: Académie de Recherche et d’Enseignement Supérieur-Commission de la Coopération au Développement, Belgique
CDD: Community-directed distributors
CDTI: Community-directed treatment with ivermectin
CI: Confidence interval
CMFL: Community microfilarial load
CRFilMT: Centre for Research on Filariasis and other Tropical Diseases, Cameroon
HD: Health district
MDA: Mass drug administration
mf/ss: Microfilariae per skin snip
NOCP: National Onchocerciasis Control Programme, Cameroon
WHO: World Health Organization
